# Paradoxical Inhibition of Glycolysis by Pioglitazone Opposes the Mitochondriopathy Caused by AIF Deficiency

**DOI:** 10.1016/j.ebiom.2017.02.013

**Published:** 2017-02-16

**Authors:** Paule Bénit, Alice Pelhaître, Elise Saunier, Sylvie Bortoli, Assetou Coulibaly, Malgorzata Rak, Manuel Schiff, Guido Kroemer, Massimo Zeviani, Pierre Rustin

**Affiliations:** a INSERM UMR 1141, PROTECT, INSERM, Université Paris Diderot, Sorbonne Paris Cité, Paris, France; b INSERM UMR 1124, Centre Universitaire des Saints-Pères, Université Paris Descartes, Sorbonne Paris Cité, Paris, France; c Reference Center for Inherited Metabolic Diseases, Hôpital Robert Debré, Assistance Publique – Hôpitaux de Paris, 48 Boulevard Sérurier, 75019 Paris, France; d Equipe11 labellisée Ligue Nationale contre le Cancer, Centre de Recherche des Cordeliers, Paris, France; e INSERM U1138, Centre de Recherche des Cordeliers, Paris, France; f Metabolomics and Cell Biology Platforms, Institut Gustave Roussy, Villejuif, France; g Pôle de Biologie, Hôpital Européen Georges Pompidou, AP-HP, Paris, France; h Karolinska Institute, Department of Women's and Children's Health, Karolinska University Hospital, Stockholm 17176, Sweden; i MRC-Mitochondrial Biology Unit, Cambridge, Cambridgeshire, United Kingdom

**Keywords:** AIFM1, X-linked mitochondrial encephalomyelopathy, Pre-clinical trial, Glyceraldehyde-3-phosphate dehydrogenase (GAPDH), Bezafibrate, Melatonin

## Abstract

Mice with the hypomorphic *AIF*-*Harlequin* mutation exhibit a highly heterogeneous mitochondriopathy that mostly affects respiratory chain complex I, causing a cerebral pathology that resembles that found in patients with AIF loss-of-function mutations. Here we describe that the antidiabetic drug pioglitazone (PIO) can improve the phenotype of a mouse Harlequin (Hq) subgroup, presumably due to an inhibition of glycolysis that causes an increase in blood glucose levels. This glycolysis-inhibitory PIO effect was observed in cultured astrocytes from Hq mice, as well as in human skin fibroblasts from patients with AIF mutation. Glycolysis inhibition by PIO resulted from direct competitive inhibition of glyceraldehyde-3-phosphate dehydrogenase (GAPDH). Moreover, GAPDH protein levels were reduced in the cerebellum and in the muscle from Hq mice that exhibited an improved phenotype upon PIO treatment. Altogether, our results suggest that excessive glycolysis participates to the pathogenesis of mitochondriopathies and that pharmacological inhibition of glycolysis may have beneficial effects in this condition.

## Highlights

• Respiratory chain-defective Harlequin mice have low glycaemia but their phenotype yet variously improved with pioglitazone.
• The phenotype improvement observed with pioglitazone in a mouse subgroup correlates with an increased glycaemia.
• Pioglitazone inhibits glyceraldehyde-3-phosphate dehydrogenase reducing exaggerated glycolysis in the Harlequin mice.

Human mitochondriopathies constitute a group of metabolic diseases with spectacularly heterogeneous clinical presentation and without efficient therapy so far. A similar variability of the disease presentation hallmarks the Harlequin mice suffering a mitochondriopathy. We analyzed mouse inter-individual response to drugs. Adopting such a “personalized medicine” approach, we identified a subgroup of responders to the anti-diabetic drug, pioglitazone. Paradoxically, the improvement of phenotype correlated with an increased glycaemia of the initially hypoglycemic Harlequin mouse. Studying this subgroup allowed us to identify the glyceraldehyde-3-phosphate dehydrogenase enzyme as an additional target of pioglitazone.

## 1 Introduction

Mitochondrial (mt) disorders represent an expanding group of diseases characterized by wide variability in clinical presentation and course (Turnbull and Rustin, 2015). Our understanding of these pathologies remains limited despite the elucidation of a huge number of the underlying gene defects. With a few exceptions (*e.g.* primary CoQ_10_ deficiency), no therapy can currently be offered to the patients and hardly any clinical trial has led to reliable and convincing conclusions (Koopman et al., 2016). This depends in part from the difficulty to collect sufficiently large cohorts of patients with a homogeneous genetic defect and similar clinical presentation. In this context, the use of animal models may provide a clue for identifying and testing therapies. However, in spite of extensive studies particularly on murine models, very few candidate drugs have shown some positive effects in subsequent human trials (Hackam and Redelmeier, 2006). A number of reasons have been advocated to account for this failure, and one of them may be disparity of clinical phenotypes between humans and mice (Rice, 2012). In addition, the extreme variability in clinical presentation and course is observed even within the same family, which suggests a relevant, albeit still unclear, role of additional genetic, epigenetic and environmental factors in the natural history of these conditions (Jain et al., 2016). Accordingly, the genetic background should be taken into account in modelling specific mt diseases (Benit et al., 2010 #4255). For instance, studies on mouse models are usually carried out on highly selected, inbred, isogenic individuals. This may be useful in the elucidation of disease mechanism or in investigating the function of a disease gene, but is inadequate to test drug efficacy in a clinically relevant (and hence heterogeneous) setting. In addition to these genetic considerations, the nursing conditions, such as cage constraint, reduced exercise, idleness, and *ad libitum* feeding, and the improper conception and execution of some studies (Couzin-Frankel, 2013), all concur to explain why so many murine models fail to yield convincing results in pre-clinical studies.

In a more than five-year-long study, we used a non-isogenic respiratory chain (RC) deficient mouse strain, namely the *Harlequin* (Hq) mouse, to test a set of drugs. We previously attributed the Hq mouse phenotype to a partial defect of RC complex I (CI) activity (Vahsen et al., 2004), due to a retroviral insertion in the X-linked *AIF* (apoptosis inducing factor) gene, leading to the formation of a hypomorphic allele (Klein et al., 2002). Noticeably, the complete inactivation of *AIF* in genetically-engineered mice (Pospisilik et al., 2007) or deleterious mutations in humans (Ghezzi et al., 2010) have a wider impact on the RC, affecting also complexes III and/or IV. The knockout, knockdown or hypomorphic mutation of the *AIF* causes a defect in CHCHD4-dependent RC biosynthesis in human cell lines in vitro, as well as in mice in vivo (Hangen et al., 2015). Similarly, mutations that occur in the human gene encoding for AIFM1 and that affect the binding of AIF to CHCHD4 cause mitochondriopathies that manifest as a severe X-linked mt encephalomyelopathy in infants (Meyer et al., 2015).

A spectacular inter-individual variability in time of onset and severity characterizes the Hq disease in the CW/BL genetic background (Bénit et al., 2008). We previously reported these variable features associated with the partial loss of CI activity in the Hq mouse and showed the positive effect of a high-fat diet on the disease course (Schiff et al., 2011). In a further attempt to identify disease-attenuating drugs in Hq mice, we selected three drugs postulated to have different mechanisms of action, being either PPAR-α (bezafibrate; BZ) or PPAR-γ (pioglitazone; PIO) agonist or having an antioxidant effect (melatonin; ML), this latter *a priori* not linked to the activation of the melatonin-specific receptor. These three drugs have been previously reported as having a potential action against mitochondriopathies, ML (Reiter et al., 2007), BZ (Wenz et al., 2008) and PIO (Pinto et al., 2016). Here, we report that PIO may improve the Hq phenotype, presumably through the inhibition of excessive glycolysis.

## 2 Materials and Methods

### 2.1 Housing and Treatment of Mice

Hemizygous Hq males (*Hq*/Y) were obtained by mating heterozygous (*Hq*/X) females with WT males obtained from the Jackson Laboratory (Bar Harbor, ME). We used F1 mice bred from founders having a mixed genetic background (B6CBACaAw-J/A-Pdcd8*Hq*/J). Mice were housed with a 12 h light/dark cycle with free access to food and water. To avoid any bias, one month-old litters were assigned to predefined (before birth) groups. Four groups were studied for melatonin (ML; Sigma M525; 4 mg/ml H_2_O) trialing (Hq or WT fed R03 pellets (control diet) *minus* or *plus* ML in bottle water; six groups for trialing bezafibrate (BZ; Sigma B7273) and pioglitazone (PIO; ChemPacific 112529-15-4) in the pellets (Hq fed R03; R03 *plus* BZ or PIO; estimated 0.925 g/kg/d and 3 mg/kg/d respectively). WT mice were fed R03, R03 *plus* BZ or *plus* PIO. We checked that WT and Hq mice consumed similar food amounts per day. Customized pellets were prepared by SAFE (Augy, France).

### 2.2 Genotype Determination

Mice were genotyped using multiplex PCR with two primers for sex determination (SRY: 59-TGGGACTGGTGACAATTGTC-39 and 59-GAGTACAGGTGTGCAGCTCT-39), two for the wild-type *Aif* allele (*Aif* 1F: 59AGTGTCCAGTCAAAGTACCGG-39; *Aif* 1R: 59-CTATGCCCTTCTCCATGTAGTT-39), and one for the *Aif* allele (*Aif* RV: 59-CCCGTGTATCCAATAAAGCCTT-39) (Bénit et al., 2008).

### 2.3 Ethics Statement

Details of the mouse study were approved by the Robert Debré-Bichat Ethics Committee on Animal Experimentation (http://www.bichat.inserm.fr/comite_ethique.htm; Protocol Number 2010-13/676-003) in accordance with the French and European Laws on animal protection.

### 2.4 Phenotype Evaluation

Locomotor ability, balance and coordination, of WT and Hq mice were assessed on a Rotarod device (Imetronic; Pessac, France). The mice were trained for 3 consecutive days after a previous days of training at 4, 5 and 6 months, at increasing speeds (4–40 rpm). The latency before fall from the rod was recorded. Dystonia was measured using the tail suspension test (Glynn et al., 2005). When suspended by its tail, a WT mouse tries to escape by splaying its hind limbs away from the trunk, while an Hq mouse holds its hind limbs against its trunk. If the hind limbs were 10 s splayed outward, it was assigned a score of 2. If one or both hind limbs were retracted for > 50% of the time, it received a score of 1. If its hind limbs were entirely and consistently retracted, it received a score of 0. Muscular strength of the forelimb was measured by a grip strength meter (Ugo Basile SRL, Varese, Italy). Each mouse was given four successive trials and a mean grip strength reading was calculated. Blood glucose was measured by a trained unique investigator between 2 and 3 pm using OneTouch Vita - glucometer (LifeScan, France) in mice with free access to food and water. Blood was collected either from the tail or from the heart at sacrifice in 6 month-old mice.

### 2.5 Mouse Astrocytes and Human Skin Fibroblasts

Astrocytes were prepared from meninges-free cerebellum of 6–7 days old mice. Astrocytes were plated into culture flasks in DMEM containing glucose (1 g/l) and 10% fetal calf serum at 37 °C in a 5% CO_2_. Upon confluence, flasks were shaken (180 rpm × 30 min; Rocking Orbital shaker, VWR) to remove contaminated microglia cells. Astrocytes are then detached from the culture flash by trypsin and pelleted at 1500 g × 5 min.

Skin fibroblasts were derived from two healthy individuals, and two patients (P1 and P2) harboring R201del missense mutations in AIF (Ghezzi et al., 2010). Fibroblasts were grown either in DMEM glucose (4.5 g/l), 6 mM glutamine, 10% FCS, 200 μM uridine, penicillin/streptomycin (100 U/ml) *plus* 10 mM pyruvate, or in selective medium (DMEM containing glutamine but no glucose, uridine nor pyruvate). For cell counting and size determination, cells were at 50% confluence (d = 0). After 5 days, cell density (cell number per cm^2^) was estimated from representative phase-contrast photographs taken on an LSM 5 Exciter optic microscope (Eclipse TE300 Nikon, France) (× 4). Cell density (cell number per cm^2^) was estimated in 54 identical areas for each condition. Cell size (length and width) was determined on the same representative photographs for a minimum of 90 cells. Width was estimated at the area adjacent to nuclei.

### 2.6 Immunocytochemistry

Immunocytochemical studies were performed on mouse cultured astrocytes adherent to coverslips (Schildge et al., 2013) and stained with Abcam primary antibodies (GAPDH ab8245; GFAP ab7260) and fluorescent-tagged secondary antibody (mouse, AlexaFluor 555 or rabbit, AlexaFluor 488; Thermofisher Scientific, France). Cells were imaged using a 20 × Plan-Apochromat (aperture 0.8)/63x EC Plan-Neofluar oil-immersion lens (aperture 1.3) of a Zeiss Axio Observer inverted microscope (Carl Zeiss, Jena, Germany) equipped with an AxioCam MRm camera.

### 2.7 Immunoblot Analyses

Western blot analysis was performed either on supernatants (200 g × 5 min) of tissue homogenates in 20 mM Tris buffer (pH 7.2), 250 mM saccharose, 40 mM KCl, 40 mM EGTA and 1 mg/ml bovine serum albumin or on cells lysed in 5 mM Tris buffer (pH 7.5), 1% Triton-X100 and 150 mM NaCl. After protein determination, extracts were separated by SDS-PAGE, and blotted onto a PVDF membrane. The membranes were blocked for 1 h in Tris-buffered saline added with 0.01% Tween-20 (TBST), 5% fat-free milk powder and incubated with primary antibodies overnight at 4 °C (AIF 1 ∶ 1000, Chemicon Merck Millipore, Darmstadt, Germany; CI 39-kDa subunit 1 ∶ 1000, Molecular Probes, Eugene, Oregon, USA; VDAC 1 ∶ 1000, ab14734; GFAP 1:10,000; GAPDH 1/5000; PPARγ 1:1000, ab27649; SOD_2_ (mitochondrial) 1:5000, ab13533; NF-ĸB phosphorylated 1:500 ab51059; Tau phosphorylated 1:5000, ab32057; NF-ĸB 1:500 Sc372). Membranes were then washed in TBST and incubated with mouse or rabbit peroxidase-conjugated secondary antibodies for 2 h at room temperature, followed by washing and visualization using ECL prime detection reagent (GE Healthcare, France) or Western Lightning Ultra (Perkin Elmer, France) and GBox imaging system (Ozyme, France). Noticeably, variances between sample signals within one given immunoblot analysis are not due to differences in exposure time, always kept constant, but rather to differences in gel loading.

### 2.8 Respiratory Chain (RC) and Antioxidant Activities

The measurement of RC activities, catalase and total (cytosolic and mt) SOD activities was carried out using a Cary 50 spectrophotometer (Varian Australia, Victoria, Australia), as described (Marklund and Marklund, 1974, Rustin et al., 1994). Protein was estimated using the Bradford assay.

### 2.9 Glucose Oxidation, Lactate Excretion and Oxygen Consumption

For glucose oxidation (aerobic glucose utilization), cultured skin fibroblasts were treated for 7 days with either dimethyl sulfoxide (DMSO; final DMSO: 1 μl/ml culture medium) or 10 μM PIO in DMSO (final DMSO: 1 μl/ml culture medium). After trypsination, cells (10^7^) were incubated for 90 min at 37 °C in 1 ml of Krebs-Ringer phosphate buffer containing 5 mM U-^14^C-glucose (11 Gbq/mmol, isotopic dilution 1/1000, Perkin Elmer) CO_2_ was recovered for 1 h in benzethonium hydroxide after stopping the reaction with 6 N sulfuric acid. The radioactive CO_2_ was counted by liquid scintillation (Ultima Gold, Perkin Elmer). Lactate excretion and respiration by intact cell suspension (mouse cultured astrocytes or human cultured skin fibroblasts) were simultaneously measured in a magnetically-stirred, 37 °C-thermostated 1 ml-quartz cell in 750 μl of a medium consisting in 0.3 M mannitol, 10 mM phosphate buffer (pH 7.2), 5 mM KCl, 10 mM MgCl_2_ using the Xenius XC spectrofluorometer (SAFAS, Monaco).

Lactate (anaerobic glucose utilization) was measured by the continuous displacement of exogenously added lactate dehydrogenase (LDH) reaction (5 IU; L1254 Sigma) in the presence of 2 mM NAD^+^, 6 IU glutamate-pyruvate transaminase (G9880 Sigma) and 17 mM glutamate. The progressive accumulation of NADH through the LDH reaction was measured by the increase of fluorescence (excitation 365 nm; emission 460 nm; NADH fluorescence quantification was ensured for each kinetic by a final addition of 4 μM NADH). The oxygen uptake by the cells was measured with an optic fiber equipped with an oxygen-sensitive fluorescent terminal sensor (FireSting O_2_, Bionef, Paris, France). The optic fiber was either fitted to an handmade cap ensuring closing of the quartz-cell yet allowing micro-injections (0.6 mm hole diameter) for concurrent measurement of oxygen uptake with lactate excretion or fitted to a magnetically stirred, 37 °C thermostated, 250 μl handmade cell.

### 2.10 Purified GAPDH Studies

The activity of purified rabbit skeletal muscle NAD^+^-dependent GAPDH (EC 1.2.1.12; G2267 Sigma) was spectrophotometrically measured (Cary 50 spectrophotometer; 340 *minus* 380 nm) in the forward direction (NADH accumulation) in 30 mM pyrophosphate buffer (pH 8.4), 0.3 mM NAD^+^, using 10 mM GAP as a substrate (Velick, 1955). The backward activity was assayed by the coupled assay with phosphoglycerate kinase (6 IU/ml; P7634 Sigma) in 90 mM triethanolamine (pH 7.5), 2 mM MgCl_2_, 2 mM ATP, 0.4 mM NADH using 7 mM phosphoglycerate as a substrate (Heinz and Freimuller, 1982). The buffering capacity of both the medium for the 5 min incubation of the GAPDH with PIO, and the measuring medium was reinforced using 1 M KH_2_PO_4_ buffer (pH 8.6), respectively to 800 mM and 92 mM final. Consistently, DMSO (final 6‰) was added when measuring GAPDH activity in the absence of PIO.

The decay of GAPDH fluorescence upon exposure to 260 nm UV irradiation (10 nm band pass) was measured at 330 nm (10 nm band pass) in a magnetically-stirred, 37 °C-thermostated 3 ml quartz cell using the SAFAS Xenius XC spectrofluorometer. Equilibrium between monomeric and polymeric (aggregated) forms of the enzyme was studied by Western blotting using the MW ladder (250–10 kDa; Precision Plus Protein Standard, Kaleidoscope, Biorad, France).

### 2.11 Statistics

Data are presented as mean ± SD for all experiments *plus* individual values when required. Statistical significance was calculated by standard unpaired *t*-test or one-way ANOVA with Bonferroni post-test correction for more than two conditions as indicated in figure legends; a *p* < 0.05 was considered statistically significant (GraphPad Prism). A paired *t*-test was applied when studying one cell culture under identical condition with the only difference being the addition of one compound.

## 3 Results

### 3.1 Effects of Melatonin, Bezafibrate and Pioglitazone on the Hq Mouse Phenotype

**Fig. 1:**
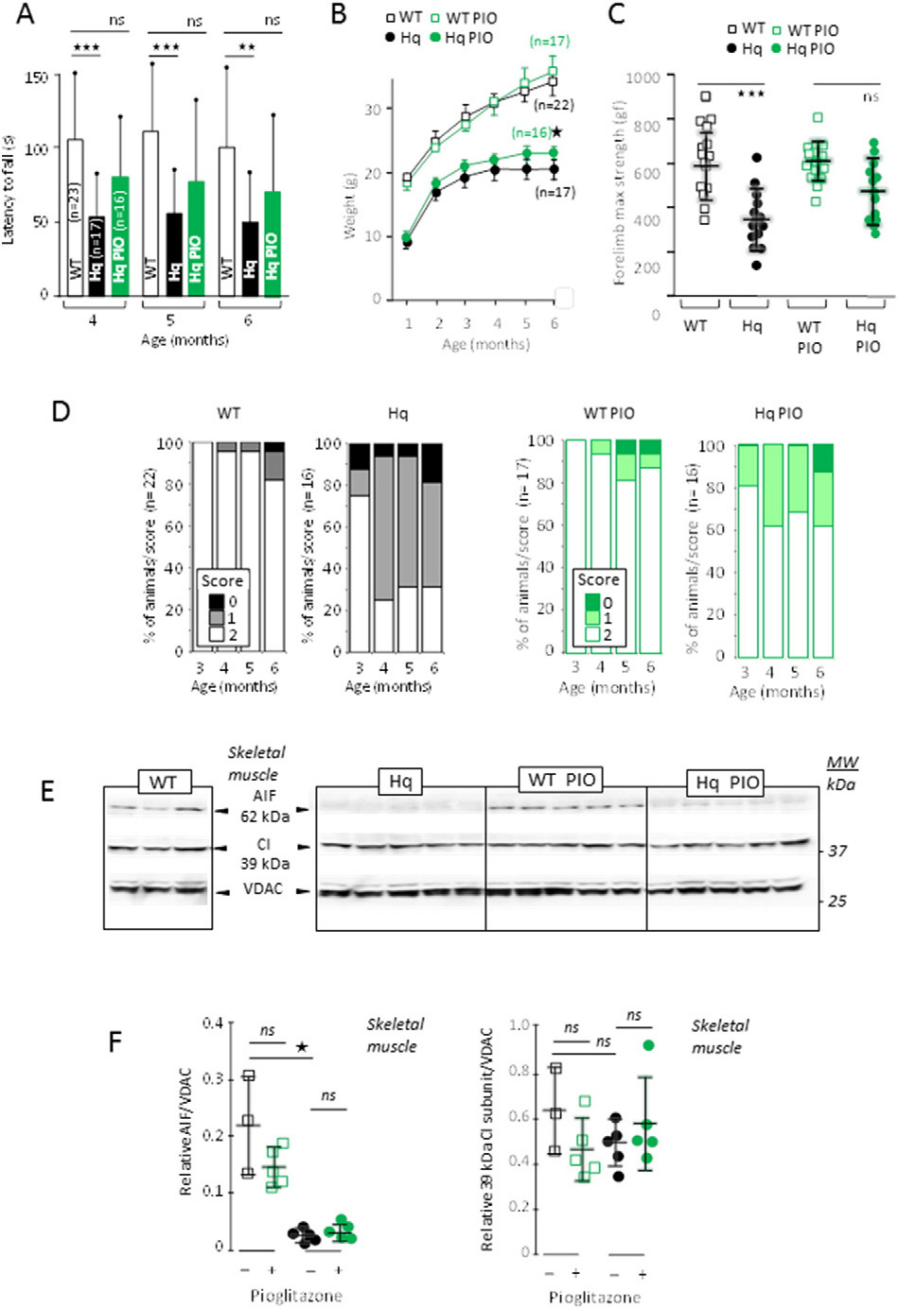
PIO improves the outcomes of the *AIF*-mutant, RC complex I deficient *Harlequin* mice. (A) Effect of PIO on motor skill (balance, coordination, overall physical condition) estimated by Rotarod test of WT (white bars), untreated (black bars) and PIO-treated (green bars) Hq mice. PIO was administrated from 1 month of age. (B) Growth curve of untreated (black open squares), PIO-treated (green open squares) WT mice and untreated (black circles) and PIO-treated (green circles) Hq mice. (C) Muscle strength of untreated WT (black open squares) or Hq (black circles) mice or PIO-treated WT (green open squares) or Hq (green circles) mice. Muscle strength was measured at 3 months of age after 2 months of treatment for treated animals. (D) Percent of untreated (left panel) WT or Hq mice or PIO-treated (right panel) WT or Hq mice as a function of their suspension test score (1–2; % of population). (E) Western blot analysis of skeletal muscle homogenates from untreated or PIO-treated 6 month-old WT and Hq mice (5 months treatment). (F) Changes in the AIF/VDAC and 39 kDa CI subunit/VDAC ratio in untreated (black open squares) and PIO-treated (green open squares) WT and in untreated (black circles) and PIO-treated (green circles) Hq mice. Mice performance evaluation, biochemical analyses and statistical tests as described under Materials and Methods.

To study the effect of melatonin (ML), bezafibrate (BZ) and pioglitazone (PIO) on the Hq mouse, we carried out a 5 months clinical monitoring initially based on the progression of ataxia assessed the Rotarod test (Bénit et al., 2008). Starting from 4 months of age untreated Hq animals generally displayed poor performance as compared to age-matched WT mice (Fig. 1A, Fig. S1A). At 4 or 5 months of age, neither ML nor BZ treatment resulted in improved score in treated Hq animals as compared to treated WT (Fig. S1A). Because ML is considered an antioxidant agent, we also tested its effects on the activities of SOD and catalase, which were previously shown to be slightly increased in the Hq skeletal muscle as compared to age-matched WT. However, we failed to detect any significant effect of ML on these two enzymatic activities (Fig. S1B). Since BZ has been recommended for the treatment of mt diseases (Wenz et al., 2008), we measured additional parameters upon BZ treatment. BZ significantly decreased the weight of WT mice, without worsening the abnormally low weight of the Hq mice (Fig. S1C). Grip tests (measuring muscle strength; Fig. S1D) and tail suspension tests (measuring neurological impairment; Fig. S1E) confirmed the inability of BZ to reduce the progression of the Hq phenotype. In line with previous reports (Watanabe et al., 1989), 5 months of BZ treatment induced liver disease in Hq mice (not shown), a side effect being presumably rodent-specific (Yatsuga and Suomalainen, 2012). Altogether, treatment with ML or BZ was found either inefficient (ML) in halting Hq disease progression or even susceptible (BZ) to worsen its course. This latter result is in line with a prior report showing that BZ can worsen the phenotype of mice that had been rendered deficient for RC by genetic engineering (Viscomi et al., 2011).

We concurrently tested the effect of the PPARγ-ligand PIO on the Hq mouse, using a low dosage (3 mg/k/d) preferable for a long term study (Biscetti et al., 2009). At 4 months of age, the Rotarod performance of Hq mice statistically differed from age-matched WT (Fig. 1A), a divergence that persisted at older ages (5, 6 months). In contrast, PIO-treated Hq mice did not statistically differ from age-matched WT mice (4, 5, 6 months of age; treatment started at 1 months of age). We also noticed that the weight of PIO-treated Hq animals slightly increased compared to untreated Hq animals, whereas the weight did not change in treated vs. untreated WT animals (Fig. 1B). A similar trend was observed at the grip test (muscle strength), so that, after 2 months of treatment, the performance of PIO-treated Hq animals was statistically indistinguishable from that of WT animals (Fig. 1C). Finally, only about 30% of untreated Hq animals scored normally at the tail suspension tails, whereas this figure rose to about 60% in the PIO-treated Hq cohort (Fig. 1D).

Altogether, these data suggested that PIO is beneficial in slowing down the progression of the mitochondriopathy of the Hq mouse. Irrespective of the test that we performed (Rotarod, grip test, tail suspension test), the response of Hq mice to PIO was however affected by a high inter-individual variability. We therefore attempted to identify the factor(s) accounting for this heterogeneity in the PIO response.

### 3.2 PIO Fails to Restore Defective AIF, CI, or to Activate Antioxidant Enzymes

**Fig. 2:**
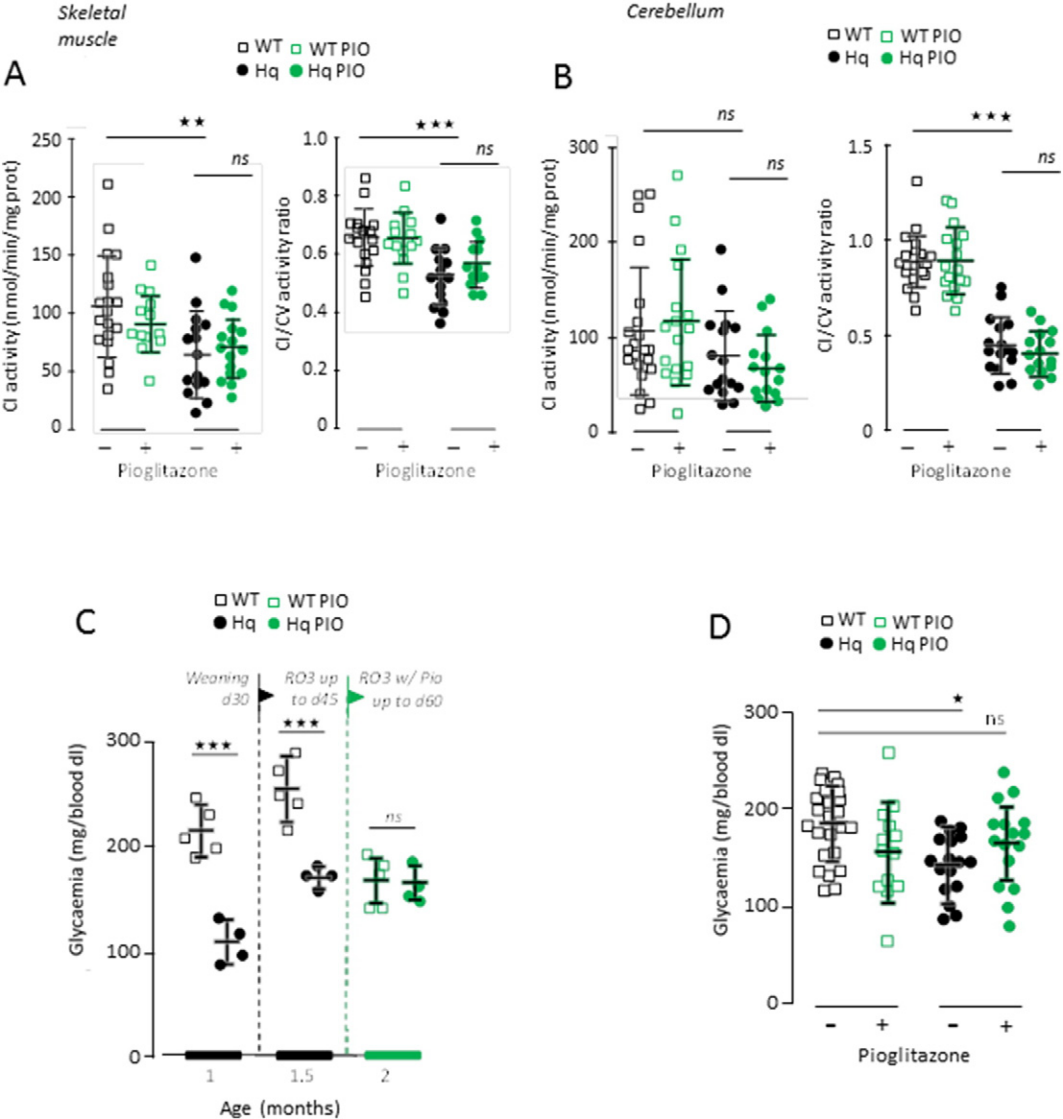
PIO lefts RC activity in the skeletal muscle and cerebellum unchanged but inversely modifies glycaemia in WT and Hq mice. (A) Activity of RC CI activity of muscle homogenate in untreated (black open squares) and PIO-treated (green open squares) WT mice and untreated (black circle) and PIO-treated (green circles) Hq mice. The activity is either expressed as mg protein (absolute activity; left panel) or ratioed to complex V (right panel). (B) CI activity of cerebellum homogenate in untreated (black open squares) and PIO-treated (green open squares) WT and untreated (black circles) and PIO-treated (green circles) Hq mice. The activity is either expressed as mg protein (absolute activity; left panel) or ratioed to complex V (right panel). (C) Bi-monthly follow-up of glycaemia of *ad libidum* fed untreated (black open square) and PIO-treated (green open square) WT and untreated (black circles) and PIO-treated (green circles) Hq mice (left panel). (D) Opposite changes of glycaemia triggered by 5 month PIO-treatment in WT (squares) and Hq (circles). Biochemical analyses, glycaemia determination and statistical tests as described under Materials and Methods.

Through its interaction with PPARγ, PIO may modulate the transcriptional landscape and induce the expression of genes coding for RC and antioxidant proteins (Morato et al., 2013, Wessels et al., 2015). Therefore, we wondered whether PIO might directly affect the expression of AIF, CI genes or favor mt biogenesis in Hq mice. Immunoblot analyses revealed that the expression of mt RC proteins was not changed in the muscle of WT and Hq mice, were they treated or not with PIO (Fig. 1E). With respect to voltage dependent anion channel (VDAC) as a reference, PIO did not rescue AIF expression in the Hq mouse muscle (Fig. 1E, F). Similarly, PIO did not increase the expression of the CI 39 kDa subunit. Accordingly, the activity of CI was not increased in the muscle from PIO-treated Hq mice, even when normalized to unaffected CV activity (Fig. 2A). Similarly, PIO failed to increase the CI activity in cerebellum from Hq mice (Fig. 2B). PIO also failed to affect the expression of Mn^2 +^-dependent superoxide dismutase (SOD2; Fig. S2A) and did not alter the enzymatic activity of total SOD or catalase (Fig. S2B, C) in the skeletal muscle of the Hq mice. Altogether, these results suggest that the positive effects of PIO on the phenotype of Hq mice are not due to direct effects on AIF expression, CI biogenesis or antioxidant systems.

### 3.3 PIO Paradoxically Decreases Glucose Utilization in AIF Deficiency

**Fig. 3:**
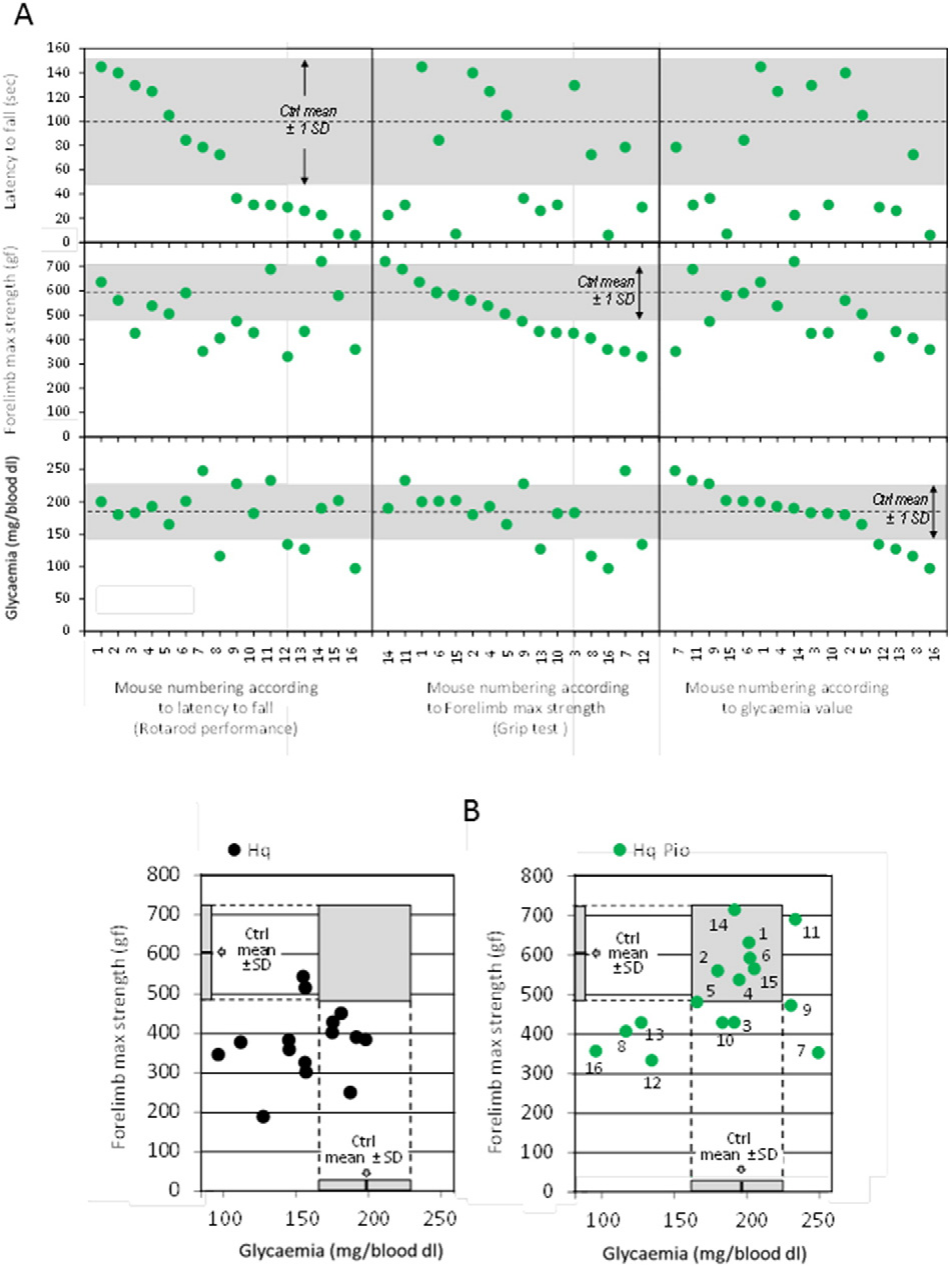
Motor coordination, muscle strength, and glycaemia of sixteen 6 month-old mice after 5 month of PIO treatment. (A) Individualized mice ranked according their motor coordination (Rotarod; upper boxes), their forelimb muscle strength (Grip test; intermediate boxes) or glycaemia (lower boxes). (B) 6 month-old Hq mice ranged according their glycaemia and their grip test performance, untreated (left panel; black circles) or 2 months PIO-treated (right panel; green circles). Numbers on the left panel refer to mouse numbering as in (A). For the sake of comparison, the mean ± 1 SD of 6 month-old untreated WT mice is indicated. Mouse performance determination and statistical tests as described under Materials and Methods.

As compared to WT mice, glycaemia was found reduced in untreated Hq mice at weaning (1 month of age; Fig. 2C). PIO is a hypoglycaemic agent (Korhonen et al., 2016) and, as expected, glycaemia was decreased after 15 days of PIO treatment in WT mice. Contrasting with this observation, glycaemia was not affected by 15 days of PIO administration in Hq mice (Fig. 2C). This phenomenon was persistent, as it was observed even after 5 months of PIO treatment, when the average value of glycaemia in the Hq mouse population became indistinguishable from that measured in untreated WT mice (Fig. 2D). At this time point, a fraction of Hq mice had blood glucose levels similar to controls (Fig. 2D). Considering either locomotor ability (Rotarod test), muscle strength (grip test), or glycaemia, a subset of Hq mice displayed values similar to those observed in untreated WT mice (Fig. 3A). Interestingly, most of the Hq mice that had normalized their glycaemia upon PIO treatment performed as WT in the grip test (Fig. 3B), an observation that was masked in the initial group analysis (Fig. 1C). Thus, it appears that the increased glycaemia induced by PIO correlates with functional improvements in Hq mice.

**Fig. 4:**
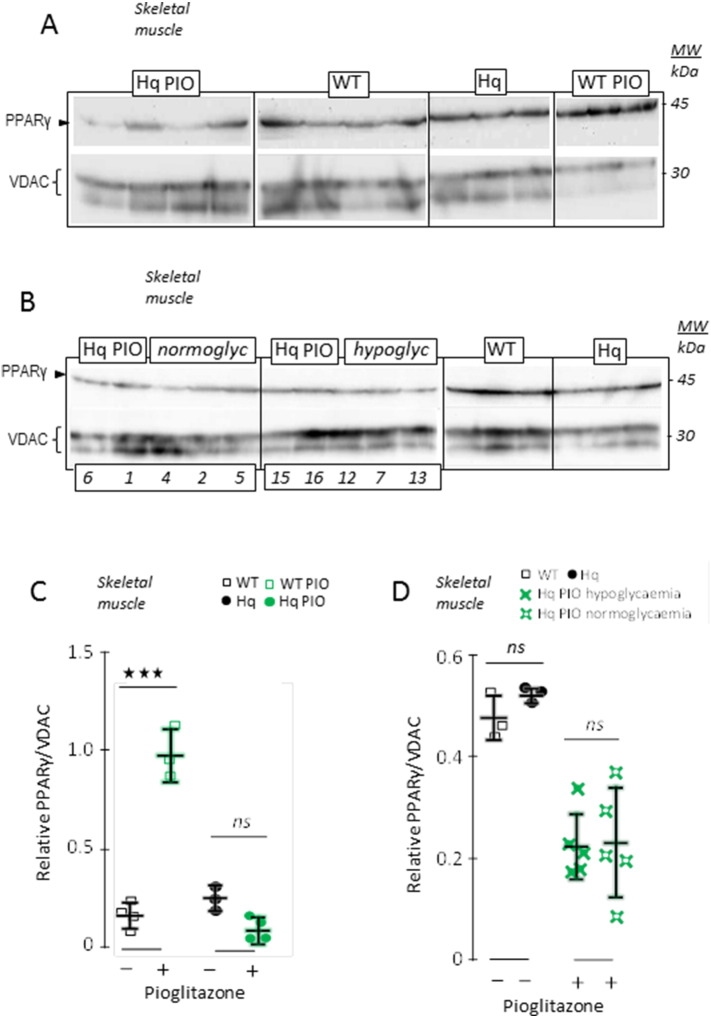
Changes in PPARγ expression do not account for glycaemia difference in the PIO-treated Hq mice. (A) Western blot analysis of PPARγ in the skeletal muscle of 6 month-old untreated and treated WT and Hq mice. VDAC was indicated as a loading control. (B) Similar analysis performed on a set of untreated WT and Hq mice, and on PIO-treated animals presented according to their normal (normoglyc) or low glycaemia (hypoglyc). Numbers on the left panels refer to mouse numbering as in (Fig. 3A). (C) PPARγ, ratioed to VDAC, in untreated (black open squares) and 5 months PIO-treated (green open squares) 6 month-old WT, and in untreated (black circles) and 5 months PIO-treated (green circles) 6 month-old Hq mice. (D) PPARγ, ratioed to VDAC, in untreated 6 month-old WT (black open squares) and Hq (black circles) mice, and in 5 months PIO-treated Hq animals pooled according to their glycaemia, low (green filled crosses) or similar to WT (green open crosses). Biochemical analyses and statistical tests as described under Materials and Methods.

We next studied the PPARγ protein (Fig. 4), a PIO-activated transcriptional activator (Garcia-Vallve et al., 2015, Gray et al., 2012), and found an increased expression in the skeletal muscle of WT mice treated with PIO (Fig. 4A). In contrast, PIO administration did not alter PPARγ protein expression in the muscle of Hq mouse (Fig. 4A, C). A similar effect of PIO on PPARγ protein was observed in the cerebellum, where it increased in WT mice, yet tended to diminish in Hq mice (not shown). No difference in PPARγ protein expression was observed between those Hq mice that presented highest glycaemia after PIO treatment and those that remained hypoglycaemic (Fig. 4B, D), meaning that the positive PIO effect on glucose levels cannot be explained by an effect on PPARγ protein levels.

**Fig. 5:**
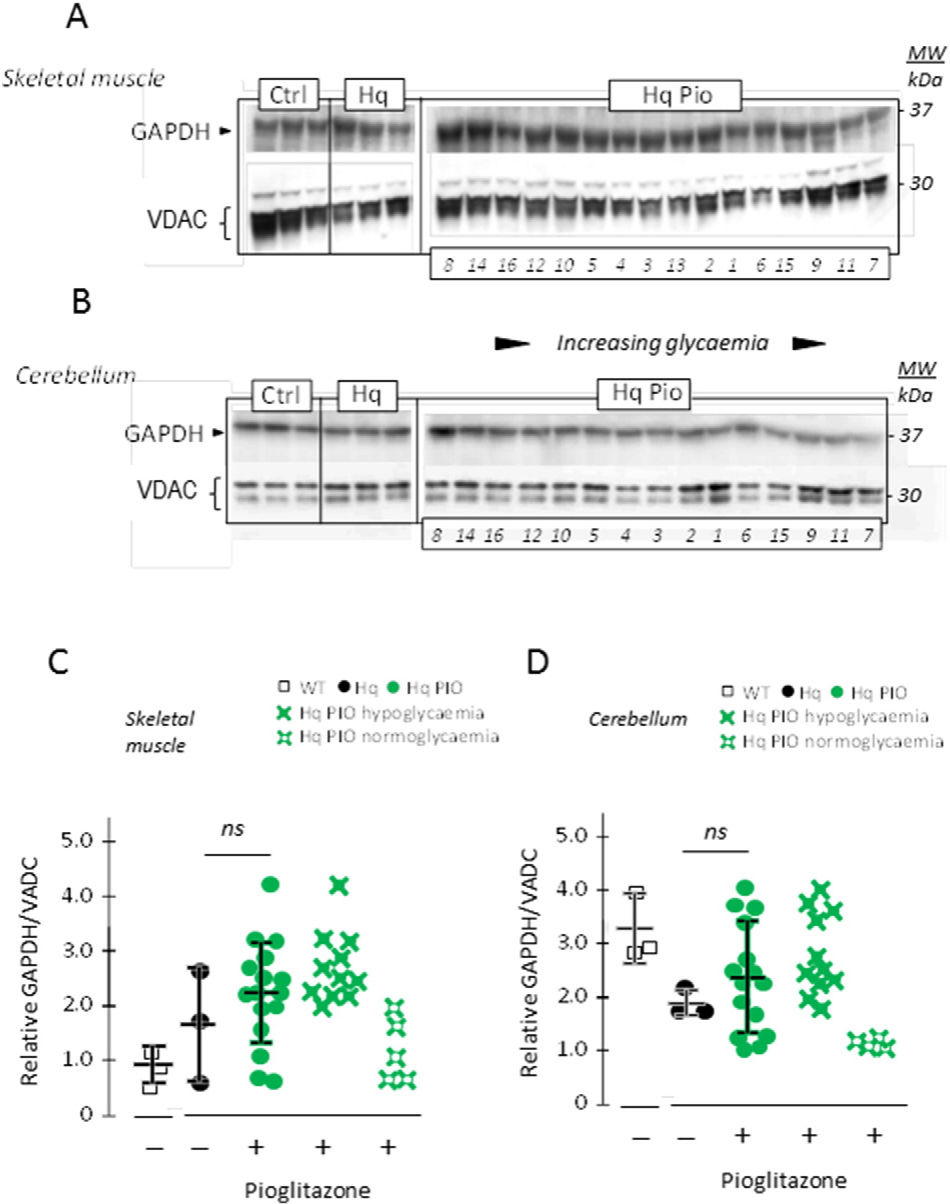
Decrease in GAPDH expression induced by PIO in the Hq mouse muscle and cerebellum can be inversely ranked according to their glycaemia status. (A) Western blot analysis of GAPDH in skeletal muscle or in (B) cerebellum homogenates from 6 month-old untreated WT, Hq mice and 5 months PIO-treated Hq mice. Protein extracts from PIO-treated mice were loaded according to their glycaemia status (ascending from the left). VDAC was indicated as a loading control. Numbers below the right panels refer to mouse numbering as in Fig. 3A. (C) GAPDH, ratioed to VDAC, in the skeletal muscle or in (D) the cerebellum of untreated 6 month-old WT (black open squares) and Hq (black circles), and in 5 months PIO-treated (green circles) 6 month-old Hq mice. PIO-treated Hq animals were also pooled according to their glycaemia, low (green filled crosses) or similar to PIO-treated WT (green open crosses). Biochemical analyses and statistical tests as described under Materials and Methods.

Among the numerous possible complications resulting from hypoglycaemia, deleterious effects on the brain have been attributed to the glycolytic enzyme glyceraldehyde 3-phosphate dehydrogenase (GAPDH) (Butterfield et al., 2010, Hildebrandt et al., 2015, Nakajima et al., 2007, Shestov et al., 2014). This enzyme can undergo diverse post-translational modifications favoring GAPDH aggregation and its translocation to the nucleus to initiate apoptotic signaling (Hara et al., 2005). GAPDH protein levels were comparable in the muscles from WT and Hq mice (Fig. 5A, C), yet tended to decrease in the cerebellum from Hq mice as compared to WT (Fig. 5B, D). PIO treatment did not result into significant changes in muscle or cerebellum GAPDH levels when considering the whole group of treated Hq mice (Fig. 5C, D). However the same Hq mice displaying high levels of GAPDH in skeletal muscle (Fig. 5A, C) and cerebellum (Fig. 5B, D) also showed low glycaemia and poor grip test performance (Fig. 3).

Considering the potentially deleterious function of GAPDH, the PIO-induced decrease of GAPDH expression in a subset of mice that exhibit improved neuromuscular function might result from, or be the cause, of favorable PIO effects. Driven by the fact that decreased GAPDH expression has been related to blunted neuroinflammation (Tristan et al., 2011), we investigated biomarkers of local inflammation including nuclear factor kappa-light-chain-enhancer of activated B cells (NF-κB), glial fibrillary acidic protein (GFAP) and Tau proteins. Because NF-κB levels were similar in WT and Hq mice (Fig. S2D), we subsequently focused on GFAP and Tau proteins. The cerebellar expression of Tau as well as that of the degraded form of GFAP were increased in Hq, yet was not affected by 5 months of PIO treatment, neither in the whole Hq group nor in the subcohort of Hq mice with the highest glycaemia values (Fig. S3). Altogether, these results suggest that the PIO-mediated GAPDH depletion does not improve the Hq phenotype due to reduced neuroinflammation. Therefore, we investigated alternative hypotheses that might link PIO-mediated GAPDH inhibition to its beneficial effects.

### 3.4 PIO is Beneficial to AIF-Deficient or Mutant Cells

To develop a culture system for investigating PIO effects at the mechanistic level, we studied PIO responses in skin fibroblasts from healthy human subjects (controls) and two patients harboring AIF loss-of-function mutations, as well as in astrocytes derived from WT and Hq mice. The two patients born from monozygotic twin sisters and unrelated fathers were previously described (Ghezzi et al., 2010) and their skin fibroblasts exhibit a severe RC deficiency (Table S1).

**Fig. 6:**
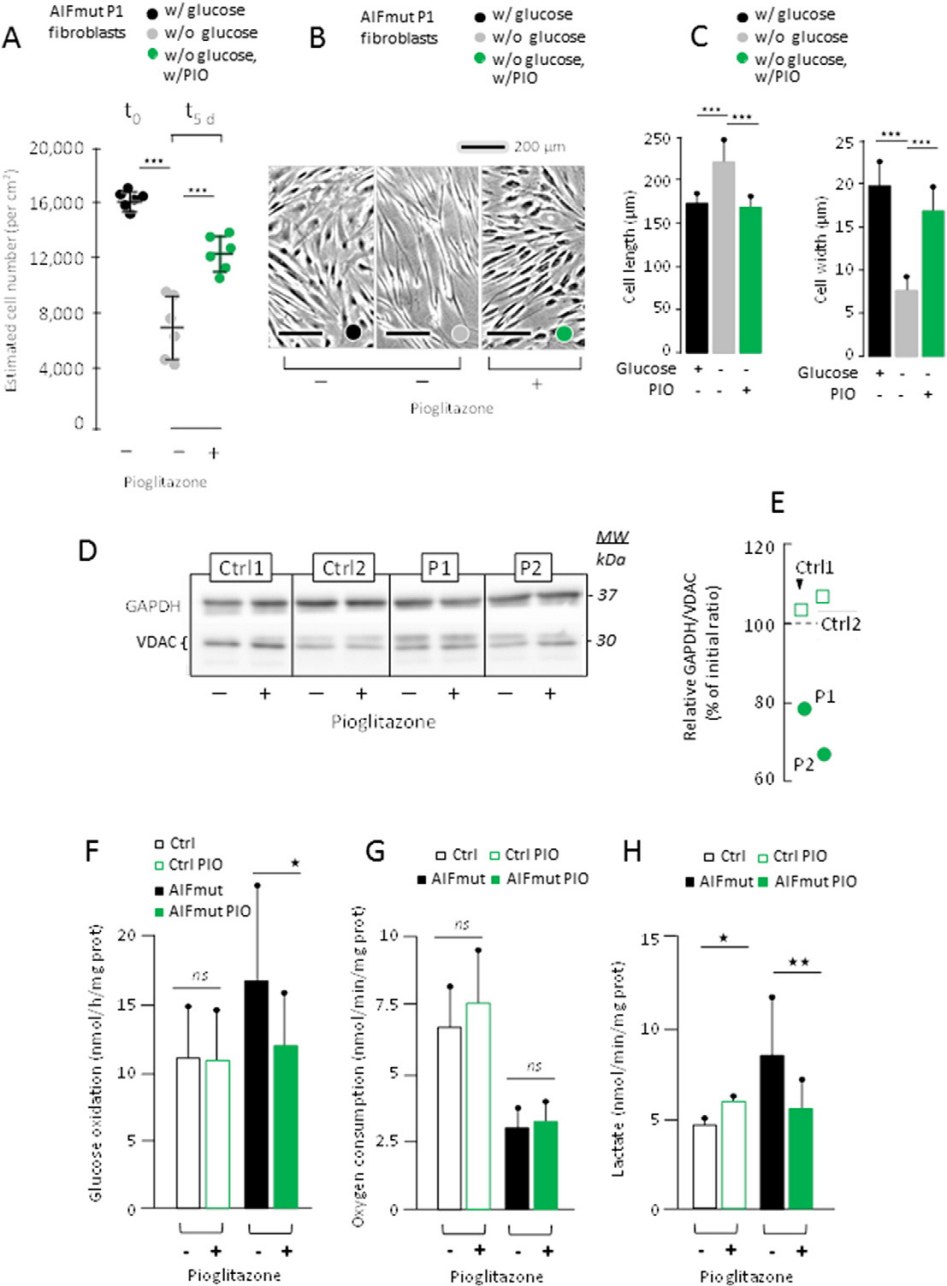
PIO-treated cultured fibroblasts derived from AIF mutant patients and grown in the absence of glucose, have decreased GAPDH expression and lower glucose utilization. (A) Effect of 5 days of 10 μM PIO treatment (0.1% DMSO final) on AIF mutant (AIFmut) P1 fibroblast number. Noticeably under the used conditions (i.e. absence of glucose), skin fibroblasts derived from control individual roughly double their number in 3–4 days depending on individual (not shown). (B) Light microscopy (× 4) of P1-derived cultured fibroblasts grown in the presence of glucose (left panel; black label), in the absence of glucose (middle panel; grey label) or in the absence of glucose but presence of 10 μM PIO (0.1% DMSO final; left panel; grey label). (C) Estimate of length (left panel) and maximal width (right panel) of P1-derived AIF mutant fibroblasts grown as in (B) in the presence (black bars), or absence (grey bars) of glucose, or in the absence of glucose but presence of PIO (green bars). (D) Effect of PIO on GAPDH expression in two controls and the two AIF-mutant P1 and P2 skin fibroblasts grown in the presence of glucose. VDAC was indicated as a loading control. (E) Effect of PIO in the presence of glucose on GAPDH/VDAC ratios in the 2 controls (Green open squares) and the P1 and P2 patients (green circles). GAPDH/VDAC ratios in the presence of PIO were expressed as a percent of initial ratio measured in the absence of PIO. (F) Mean ± 1 SD of C^14^ glucose oxidation, oxygen uptake (G), and lactate excretion (H) by control skin fibroblasts in the absence (black open bars) or presence (green open bars) of PIO or by AIF-mutant patient fibroblasts in the absence (black bars) or presence (green bars) of PIO. Cells counting, cell length and width estimation, biochemical analyses and statistical tests as described under Materials and Methods.

When seeded at similar density (1·10^6^/75 cm^2^), the absence of glucose resulted in the death of about half of AIF mutant fibroblasts after 5 days yet did not compromise the survival of control cells (Fig. 6A). The residual AIF mutant cells exhibited an altered morphology with an elongated and slender appearance (Fig. 6B and C). Adding 10 μM PIO largely protected AIF mutant cells from death (Fig. 6A) and maintained their morphology normal throughout a 20-day-long culture period (Fig. 6B and C). This effect of PIO was not due to an inhibition of mt pyruvate oxidation recently reported for various thiazolidinediones including PIO metabolites (Colca et al., 2013), as neither respiration nor mt pyruvate oxidation measured in permeabilized control and AIF mutant fibroblasts (Fig. S5). We also considered the reported effect of PIO on reverse lactic dehydrogenase reaction (i.e. the conversion of pyruvate to lactate) (Masciopinto et al., 2012). However, assay of the enzyme activity in both backward and forward directions failed to reveal any effect of the in vitro supplementation of PIO to cell cultures (not shown). Nonetheless, PIO treatment resulted in a partial reduction in GAPDH protein levels in AIF mutant fibroblasts, not in control cells (Fig. 6D, E). In keeping with what we observed in mice, PIO slightly increased GAPDH protein levels in WT (+ 5 to 10%; relative to VDAC) but decreased the expression of GAPDH in fibroblasts from AIF-mutant patients (− 20 to − 35%; Fig. 6E). Similarly, addition of PIO reduced GAPDH expression in astrocytes cultures from Hq mice (Fig. S4A–C). Based on this observation, we next investigated whether PIO would inhibit glycolysis in AIF-mutant or AIF-deficient cells.

### 3.5 PIO Inhibits Glycolysis in AIF Deficient Cells

We first noticed that C^14^-glucose was more actively oxidized by AIF mutant fibroblasts than by control cells, and that PIO normalized this excessive glucose oxidation by patient fibroblasts without affecting controls (Fig. 6F). In contrast, PIO did not improve the reduced oxygen uptake by AIF mutant fibroblasts (Fig. 6G). We next studied the effect of PIO on lactate excretion by fibroblasts. Lactate excretion was increased by PIO in control cells whereas it was decreased in AIF mutant cells (Fig. 6H). A similar trend, although less marked, was observed when simultaneously studying respiration and lactate excretion by cultured astrocytes obtained from WT or Hq mice (Fig. S4D, E). Collectively, these data indicate that PIO reduced glycolysis in AIF-deficient cells and that these effects are direct (because they are observed in cultured cells) rather than indirect (in which case they only would have been observed in vivo in mice).

### 3.6 PIO Inhibits the Enzymatic Activity of GAPDH

**Fig. 7:**
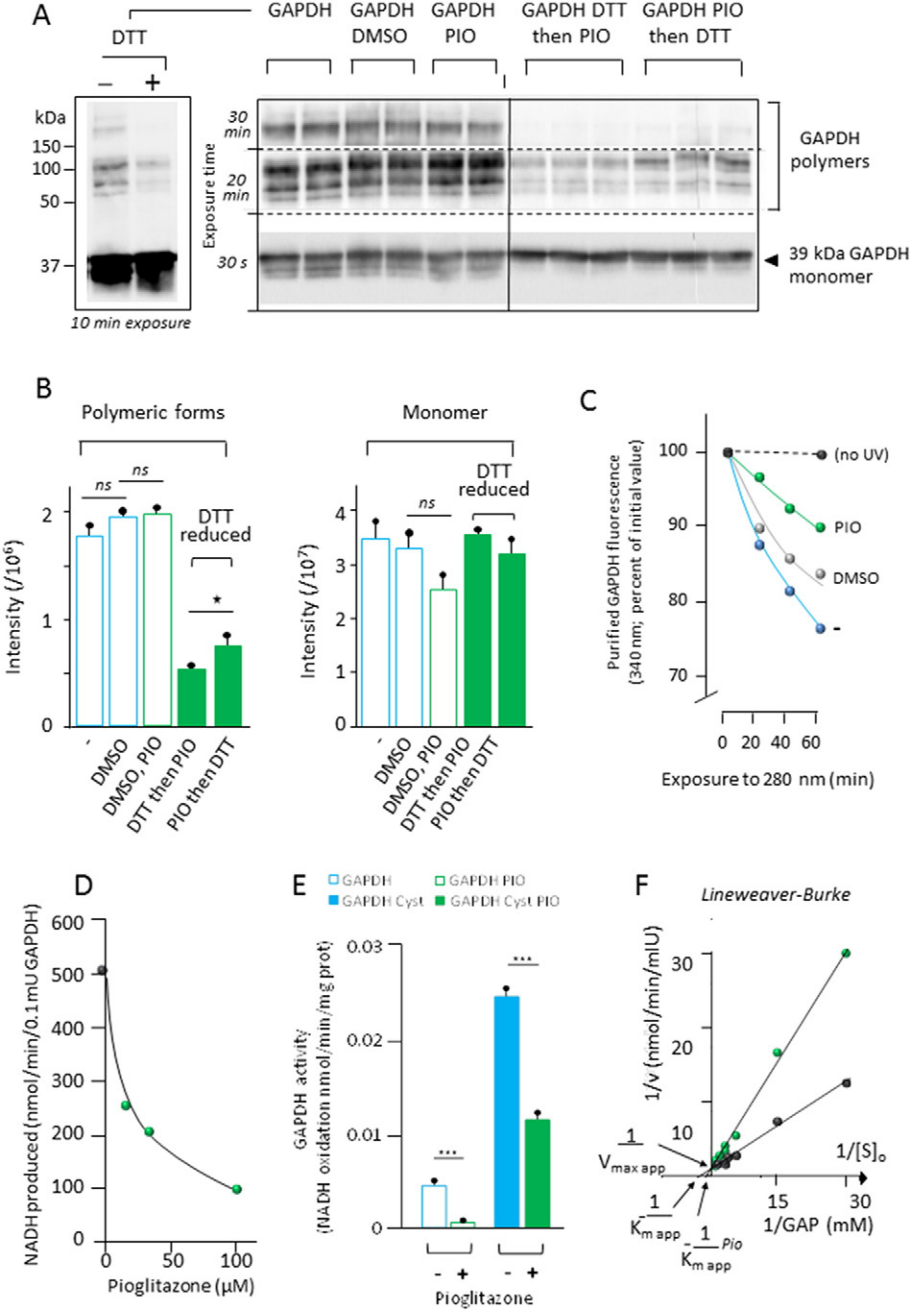
PIO interacts with and inhibits purified GAPDH. (A) Western blot analysis of purified GAPDH in the absence (left panel) or presence (right panel) of added dithiothreitol (DTT). Noticeably, no higher aggregation forms were detected in the stacking gel. (B) Quantities of different aggregation forms (tetra-, tri-, di-, and monomeric) in the absence of DTT (open bars: initial GAPDH suspension, GAPDH *plus* DMSO, GAPDH *plus* DMSO-solubilized PIO, or in the presence of DTT (green filled bars) added before or after PIO). (C) GAPDH (2.1 mIU/ml) fluorescence in the absence (blue dots) or presence (green dots) of 12.5 μM PIO against UV (280 nm) irradiation. The presence of 1.25‰ DMSO had *per se* a partial protecting effect against UV (grey dots). No changes in fluorescence were observed when the enzyme was kept away from UV irradiation (black dots). Noticeably initial fluorescence in the presence or absence of PIO, or DMSO was not different ruling out potential interference of these compounds with fluorescence assay conditions (excitation: 280 nm, 10 nm bandpass; emission: 330 nm, 10 nm bandpass). (D) Initial activity of rabbit skeletal muscle GAPDH (0.05 mIU/ml) measured in the forward direction (NADH accumulation) (Velick, 1955) and of increasing amounts of PIO. (E) Activity measured in the backward direction (NADH oxidation) in the absence (blue open bar) or presence (green open bar) of 200 μM PIO, and in the presence of 1.7 mM cysteine and absence (blue filled bar) or presence (green filled bar) of 200 μM PIO. (F) Lineweaver-Burk plots of forward GAPDH activity (0.075 mIU/ml) in the absence (black line) or presence (green line) of 62.5 μM PIO. GAP, d-glyceraldehyde 3-phosphate.

PIO modified the polymerization of purified GAPDH in vitro (Fig. 7A, B) that is known to be affected by GAPDH redox status (Nakajima et al., 2007). Under denaturing conditions, GAPDH was composed by a mixture of monomers, dimers, trimers and tetramers. Addition of dithiothreitol (DTT) reduced most of the polymeric forms of GAPDH to monomers, and this effect was attenuated by prior treatment with PIO (Fig. 7A, B). Taking advantage of the sensitivity of GAPDH to the oxidizing effect of UV light (Maloletkina et al., 2012, Yamaguchi et al., 2003) we investigated whether PIO might affect the stability of the enzyme. The fluorescence of the enzyme at 330 nm rapidly decays upon illumination with 280 nm monochromatic light, and this decay was significantly attenuated in the presence of PIO (Fig. 7C). Finally, we established that PIO reduced both the forward and backward GAPDH activities in a cell-free system (Fig. 7D, E). This inhibition amounted to > 80% in the absence of cysteine and to ~ 50% in the presence of cysteine, which acts an endogenous GAPDH activator (Velick, 1955) (Fig. 7B). Finally, we established that PIO behaves as a competitive inhibitor of GAPDH, reducing the affinity of the enzyme for its substrate, glyceraldehyde-3-phosphate (and hence enhances the apparent Km) without changing the V_max_ of the reaction (Fig. 7C). Altogether, these results suggest that PIO can directly interact with GAPDH, thus influencing its redox biochemistry and interfering with its enzymatic function.

## 4 Discussion

While prenatal diagnosis can now be offered to ever more families presenting at risk for a genetic mt disease, no effective cure exists for these conditions (Viscomi et al., 2015). The development of rational therapeutic strategies (apart from gene therapy) is hampered by our limited understanding of the pathogenic mechanisms underlying most of these diseases. Indeed they may or may not be associated with ATP shortage, poor oxygen handling, metabolic imbalance, abnormal programmed cell death or conversely proliferation, this depending on each mutation in specific gene, possibly varying in different organs or cell types. In this context, we decided to investigate a set of three drugs, each potentially acting on mt functions, on a preclinical model of RC defective mice, the Hq strain. Based on a first 6-month-long trial using 163 Hq mice, we left aside melatonin and bezafibrate, showing no positive effect in our assay conditions, to focus on PIO, which improved several traits of the Hq phenotype. This observation was possible despite the choice to study a limited number of genetically distinct individuals harboring the Hq mutation (about 15 individuals per condition). Nonetheless, we observed that most individuals were poorly performing upon PIO treatment. Only a few PIO-treated Hq mice improved their neuromuscular performances reaching WT values. This inter-individual variability in the PIO response was observed in the F_1_ mice bred from founders having a mixed genetic background (B6CBACaA^w-J^/A-Pdc8^*Hq*^/J) housed under identical conditions. Such a heterogeneous response to treatment is not an unusual observation in mt diseases. It has been reported for idebenone treatment of Leber Hereditary Optic Neuropathy (which results from CI-deficiency) (Klopstock et al., 2013) and Friedreich ataxia (which results from reduced CI, CII and CIII functions secondary to a generalized deficiency of the iron-sulfur proteins) (Schiff and Rustin, 2016). In keeping with this, Bayesian analyses of patient's scores measuring performance on different clinical scales (ICARS, FARS or SARA) failed to disclose statistically significant effect of PIO on Friedreich ataxia patients (Andriss et al., 2015). However, similarly to our pre-clinical study on PIO-treated Hq mice, when individual scores of treated vs untreated FRDA patients were considered, a sub-group of patients (about 20%) manifested a consistent improvement (2 year trial) irrespective to the evaluation scale. Because of this wide inter individual response to therapy observed in mt diseases, we thus recommend that trials should stratify the results according to the possible existence of responders vs. non-responders categories. Our results corroborate the idea that pharmacological trials against mt disease can be fully valid even if carried out on a limited number of patients, provided that the need for personalized stratification is taken into account. They predict that lack of careful and personalized data analysis of clinically heterogeneous patient groups (even though the diagnosis of their RC defect makes them appear molecularly homogeneous) will lead to further failures in clinical trials. Thus, we need to identify biomarkers that allow recognizing the minority of patients that may profit from pharmacological treatments.

A second interesting conclusion that can be drawn from our work concerns the paradoxical effect of PIO on Hq versus WT mice. PIO, a widely used hypoglycaemic drug, acted as predicted for a PPARγ ligand in WT mice, therefore decreasing glycaemia and weight, without significantly affecting motor performance. Conversely, the blood glucose levels, which were sub-normal in untreated Hq mice, increased under PIO treatment with the result that several Hq mice displayed blood glucose levels similar to WT. Incidentally, hypoglycaemia is an inconsistent feature of RC deficiency in humans, but low glycaemia is seldom mentioned in RC deficient mice. Yet, the ablation of *TFAM* in the mouse skeletal muscle results in RC defect, decreased blood glucose, increased glucose tolerance and insulin-independent skeletal glucose uptake (Wredenberg et al., 2006). Interestingly enough, most of the PIO treated Hq mice that normalized their glycaemia also restored their muscle strength to control values. The paradoxical effect of PIO on glycaemia of Hq mouse may be tentatively ascribed to a reduction of glucose utilization resulting from the inhibition of the glycolytic enzyme GAPDH. Targeting of GAPDH by PIO was confirmed by in vitro experiments involving astrocytes derived from Hq mice or cultured fibroblasts derived from two patients harboring loss-of-function mutations affecting the RC-sustaining function of AIF. Beside its interaction with the PPARγ receptor (Dovinova et al., 2013, Gray et al., 2012), additional PIO targets have been reported at the level of pyruvate metabolism (Divakaruni et al., 2013, Ye et al., 2016). Nonetheless, none of these putative targets appeared to mediate the beneficial effects of PIO on blood glucose levels in Hq mice. Instead, GAPDH turns out as a supplemental target that is directly inhibited by PIO in vitro and that is downregulated in Hq mice treated with PIO in vivo, as well as in cultured astrocytes from Hq mice or AIF-mutant fibroblasts exposed to PIO in vitro.

The genetic diversity of the Hq mice presumably gave us the opportunity to recognize PIO Hq responders, and to ultimately identify GAPDH as one additional PIO target. With the aim to further document the effect of PIO in RC-deficient mice, the isolation of PIO-responsive congenic Hq strains and/or the identification of PIO-responsive alternative RC-deficient mice can be considered in future years. The positive effect of PIO observed in Hq mice is reminiscent of the neuroprotection conferred by PIO in humans, in which PIO reportedly lowers the incidence of dementia among non-diabetic individuals (Heneka et al., 2015) and reduces neuroinflammation in multiple sclerosis (Negrotto et al., 2016). Considering these factors, the potential effect of PIO on patients affected by mt disease should be examined. Biomarkers that may guide the inclusion and maintenance of patients in such trials evaluating PIO may include blood glucose levels, as well as the modulation of GAPDH expression in peripheral tissues.

## Funding Sources

This work was supported by French (ANR AifInter to PR and GK) (ANR-11-BSV1-0017) and European (E-rare Genomit to PB and PR) (16-CE18-0010-02) institutions, and patient's associations to PB and PR: Association Française contre les Myopathies (AFM; Project No. 11639), Association d'Aide aux Jeunes Infirmes (AAJI), Association contre les Maladies Mitochondriales (AMMi), Association Française contre l'Ataxie de Friedreich (AFAF), and Ouvrir Les Yeux (OLY).

## Conflicts of Interest

We confirm that there are no known conflicts of interest associated with this publication and there has been no significant financial support for this work that could have influenced its outcome.

## Author Contributions

P.B. and P.R. conceived the project. G.K., M.Z., P.B., and P.R. wrote the manuscript. P.B., A.P., E.S., S.B., A.C., M.R., M.S., and P.R. conducted research. All authors contributed to data analysis and manuscript preparation.

## Appendix A Supplementary data

Supplementary material Image 1
