# Supplementary material for: Paradoxical Inhibition of Glycolysis by Pioglitazone Opposes the Mitochondriopathy Caused by AIF Deficiency
Source: eBioMedicine. 2017 Feb 16;17:75–87. doi: 10.1016/j.ebiom.2017.02.013 (PMC5360583; doi:10.1016/j.ebiom.2017.02.013)
Supplement: Supplementary file 1 — Supplementary material [file mmc1.pdf]

SUPPLEMENTAL INFORMATIONS

SUPPLEMENTAL FIGURES AND TABLE

Fig. S1. Effect of melatonin and bezafibrate on WT and Hq mice.

(A) Motor skill estimated by Rotarod test of WT (white bars; n=17), untreated (black bars; n=13), melatonin (ML)-treated (orange bars; n=12) and bezafibrate (BZ)-treated (red bars; n=17) *Harlequin* (Hq) mice at 4, and 5 month of age. Drugs were administrated at 1 month of age. (B) Total SOD and catalase activities, measured in skeletal muscle homogenate of 7 month-old untreated (open black squares) or treated WT mice (open orange squares; 6 months treatment) and 7 month-old untreated (black circle) or treated Hq mice (orange circle). (C) Growth curve of untreated (open black squares; n=17) and BZ-treated WT mice (open red squares; n=17) and untreated (black circle; n=13) and BZ-treated (red circle; n=17) Hq mice. (D) Muscle strength of untreated WT mice (black open bar; n=17) or Hq mice (black dark bar; n=13) and BZ-treated WT (red open bar; n=15) or Hq (red dark bar; n=12). Muscle strength was measured at 3 months of age after 2 months of treatment for treated animals. (E) Percent of WT (left panel; n=22) and Hq (right panel; n=17) mice and BZ-treated WT (left panel; n=17) and Hq (right panel; n=14) mice as a function of their suspension test score (1 to 3; % of population). Drug posology, mice performance evaluation, biochemical analyses and statistical tests, as described under Material and Methods.

Fig. S2. Pioglitazone does not affect antioxidant enzymes or NFκB in WT or Hq mice.

(A) Western blot analysis of mitochondrial SOD_2 in skeletal muscle of untreated WT, Hq and PIO-treated Hq mice. SOD_2 expression changes in untreated WT mice (black open squares) and untreated (black circles) or PIO-treated (green circles) Hq mice (right panel). (B) Total SOD (left panel) and (C) catalase (right panel) activity in skeletal muscle of WT mice (black open squares) and untreated (black circles) or PIO-treated (green circles) Hq mice. (D) Western blot analysis of non-phosphorylated (NP; upper panel) and phosphorylated (P; middle panel) NFκB in the cerebellum of untreated WT and Hq mice. Lower right panel, NFκB expression changes the cerebellum of WT (black

open squares) and Hq (black circles) mice. Biochemical analyses and statistical tests as described under Material and Methods.

Fig. S3. Changes in Tau, phosphorylated-Tau, GFAP and its degradation products do no account for the beneficial effect of PIO in a subset of the Hq mice.

(A) Western blot analysis of Tau, phosphorylated Tau (P-Tau), VDAC, GFAP and degraded GFAP in the cerebellum of 6 month-old WT, Hq mice and five Hq mice with increased glycaemia value (normoglyc) after 5 months of PIO treatment. (B) Changes in Tau, phosphorylated-Tau, GFAP expression relative to VDAC in the cerebellum of 6 month-old WT (black open squares), Hq (black circles) mice and five Hq mice with increased glycaemia values after 5 months of PIO treatment (open green crosses). Biochemical analyses and statistical tests as described under Material and Methods.

Fig. S4. Pioglitazone affects glycolysis in astrocytes derived from Hq mice.

(A) Western blot analysis of GAPDH and VDAC in four cultures (Hq I, II, III and IV) of untreated or PIO-treated astrocytes derived from Hq mice. (B) Changes in GAPDH ratioed to VDAC in untreated (black circles) or PIO-treated (green circles) these four astrocyte cultures. Paired test revealed a significant difference between untreated and PIO-treated cultures. (C) Typical examples of GAPDH (upper panel; x 63) expression as visualized in cultured astrocytes through immunohistochemistry carried out on WT (left; white square), or Hq astrocytes, untreated (middle; black circle) or after PIO treatment (right; green circle). Similar study (lower panel; x 20) carried out for GFAP, with nuclei stained with DAPI. (D) Oxygen consumption and (E) lactate excretion by untreated (black open bars; n=16) or PIO-treated (green open bars; n=16) WT astrocytes, and untreated (black dark bars; n=12) or PIO-treated (green dark bars; n=12) Hq mice. Biochemical analyses and statistical tests as described under Material and Methods.

Fig. S5. Pioglitazone does not affect respiration or mitochondrial pyruvate oxidation rates by cultured skin fibroblasts.

Respiration by intact skin fibroblasts untreated (black) or treated (green) by plioglitazone. Cells were subsequently made permeable to pyruvate by addition of digitonin. A limited amount of malate was added as to allow recycling of the coenzyme A necessary for a sustain oxidation of pyruvate. Maximal rate of pyruvate oxidation was obtained by the addition of the uncoupler, *m*-ClCCP. Fibroblasts were treated for 7 d with either DMSO (final DMSO: 1µl/ml culture medium) or 10 µM PIO in DMSO, under conditions where PIO affected lactate excretion (Fig. 6H).

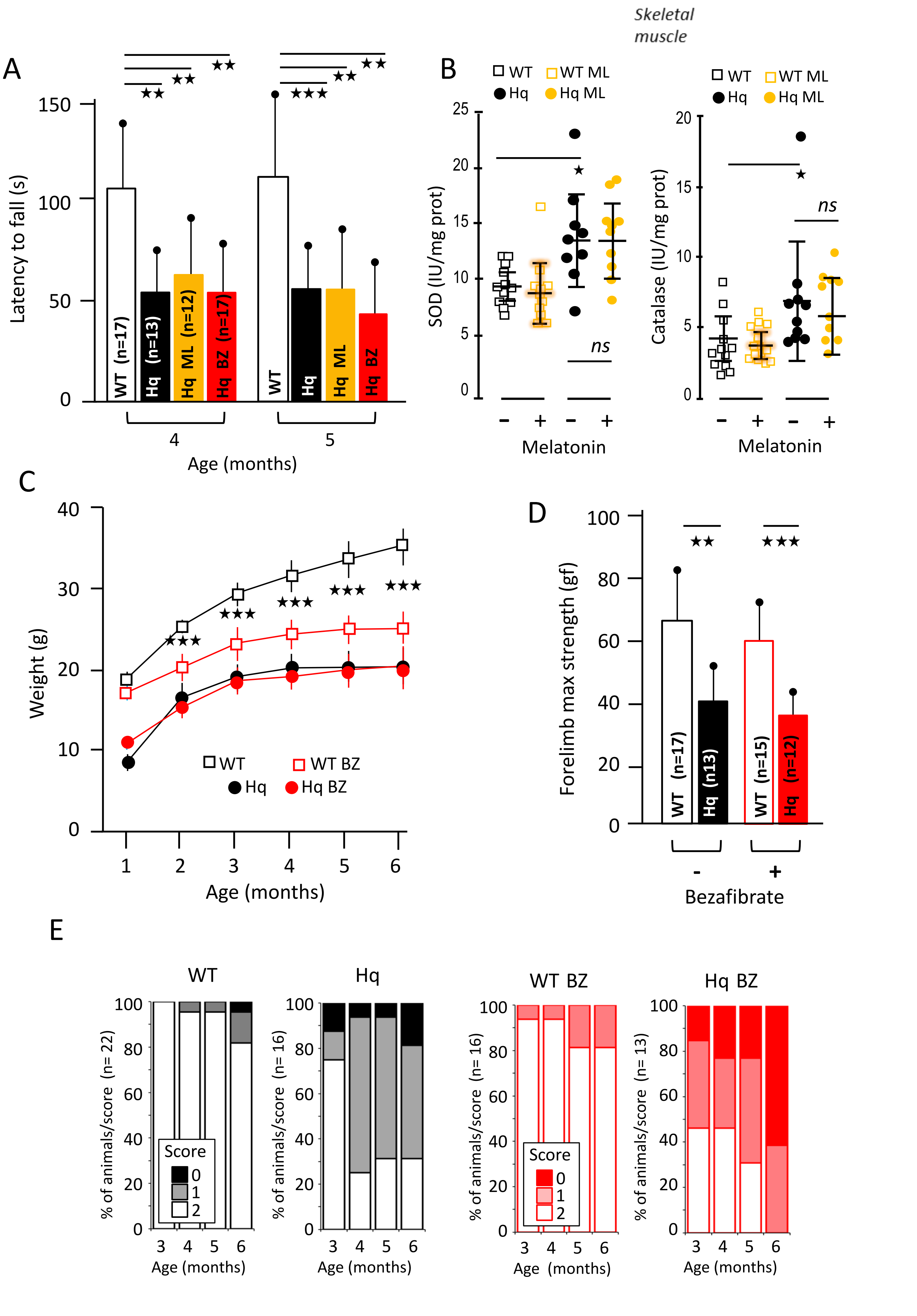

Bénit et al. Suppl Fig. 1

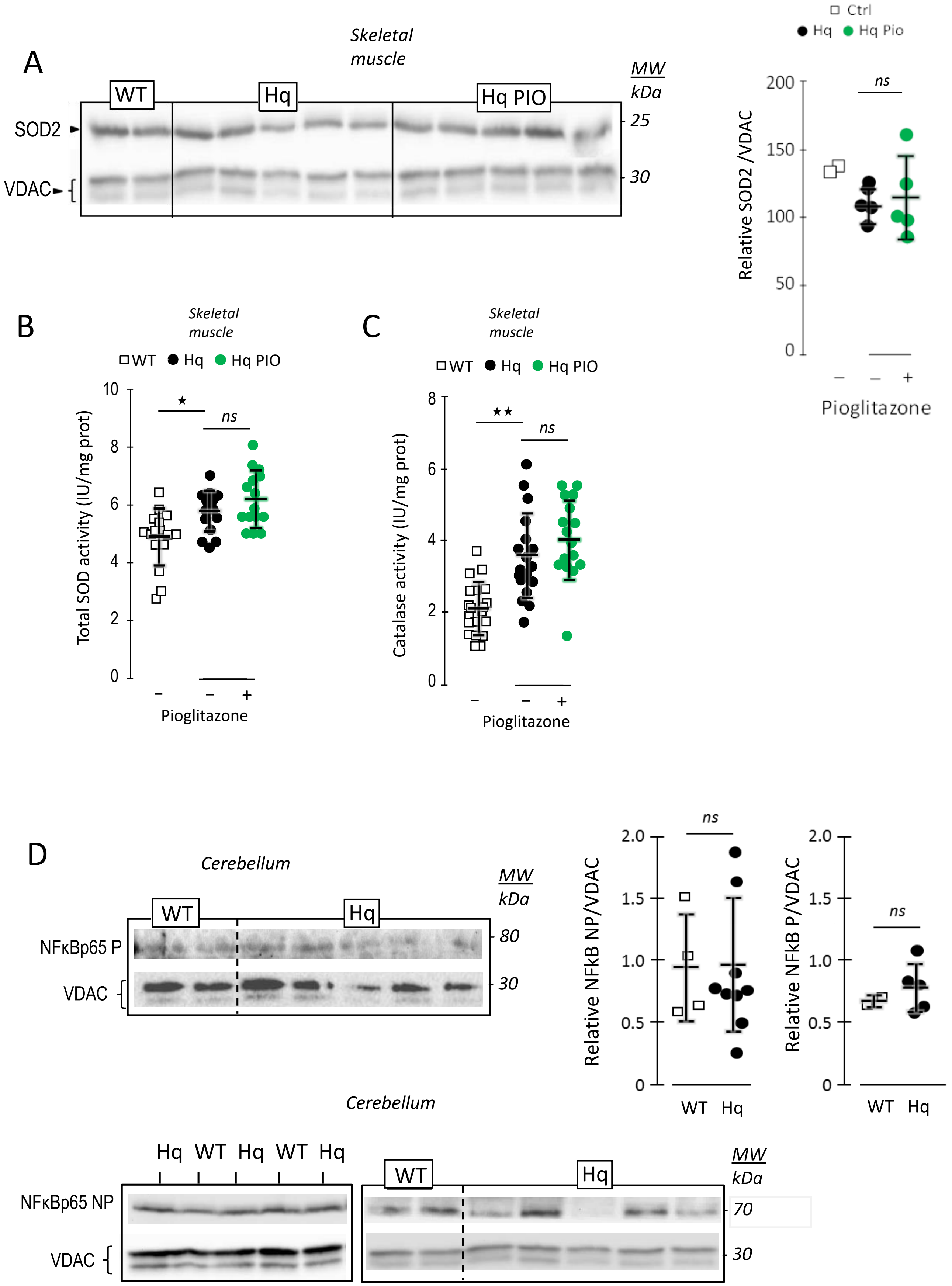

Bénit et al. Suppl Fig. 2

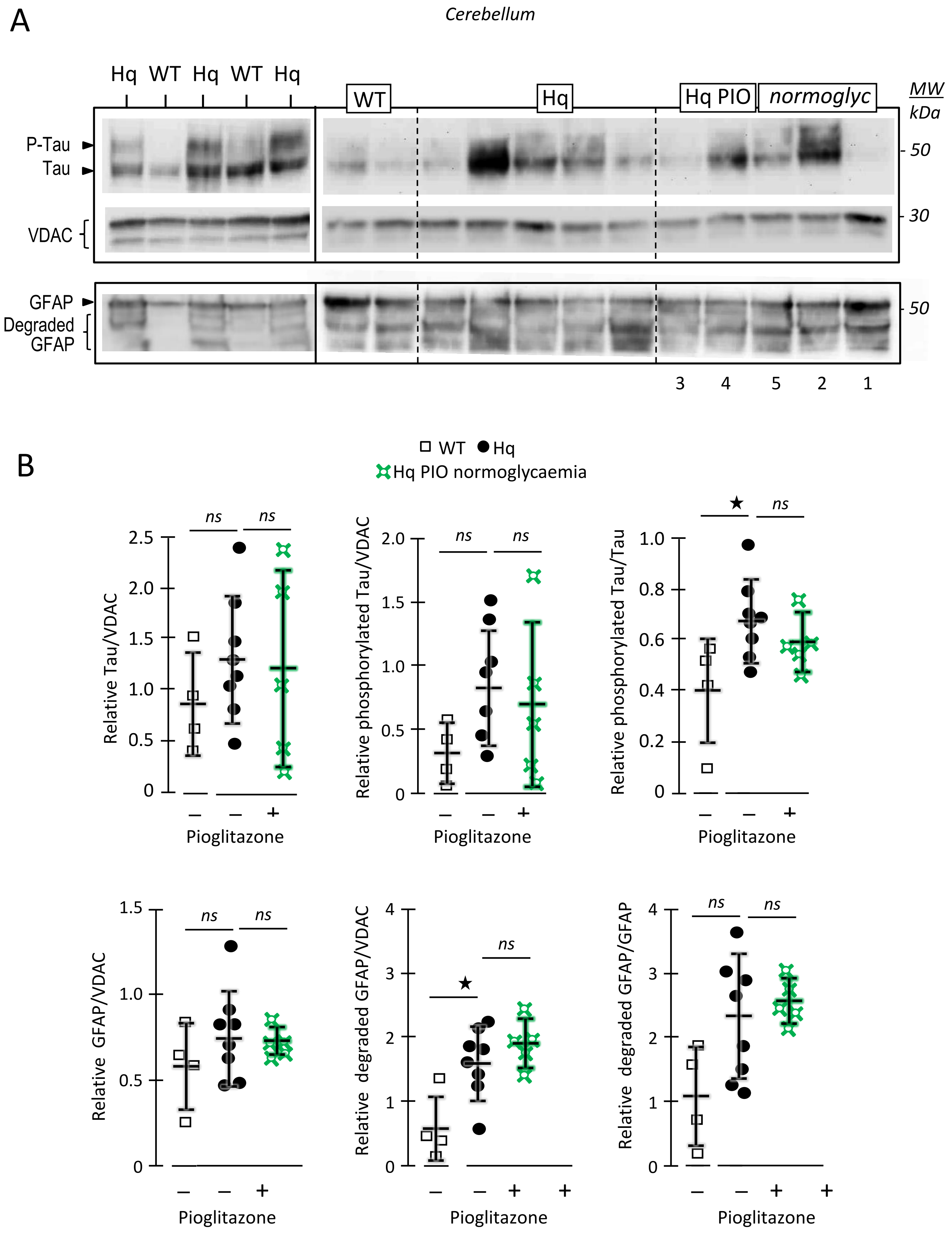

Bénit et al. Suppl Fig. 3

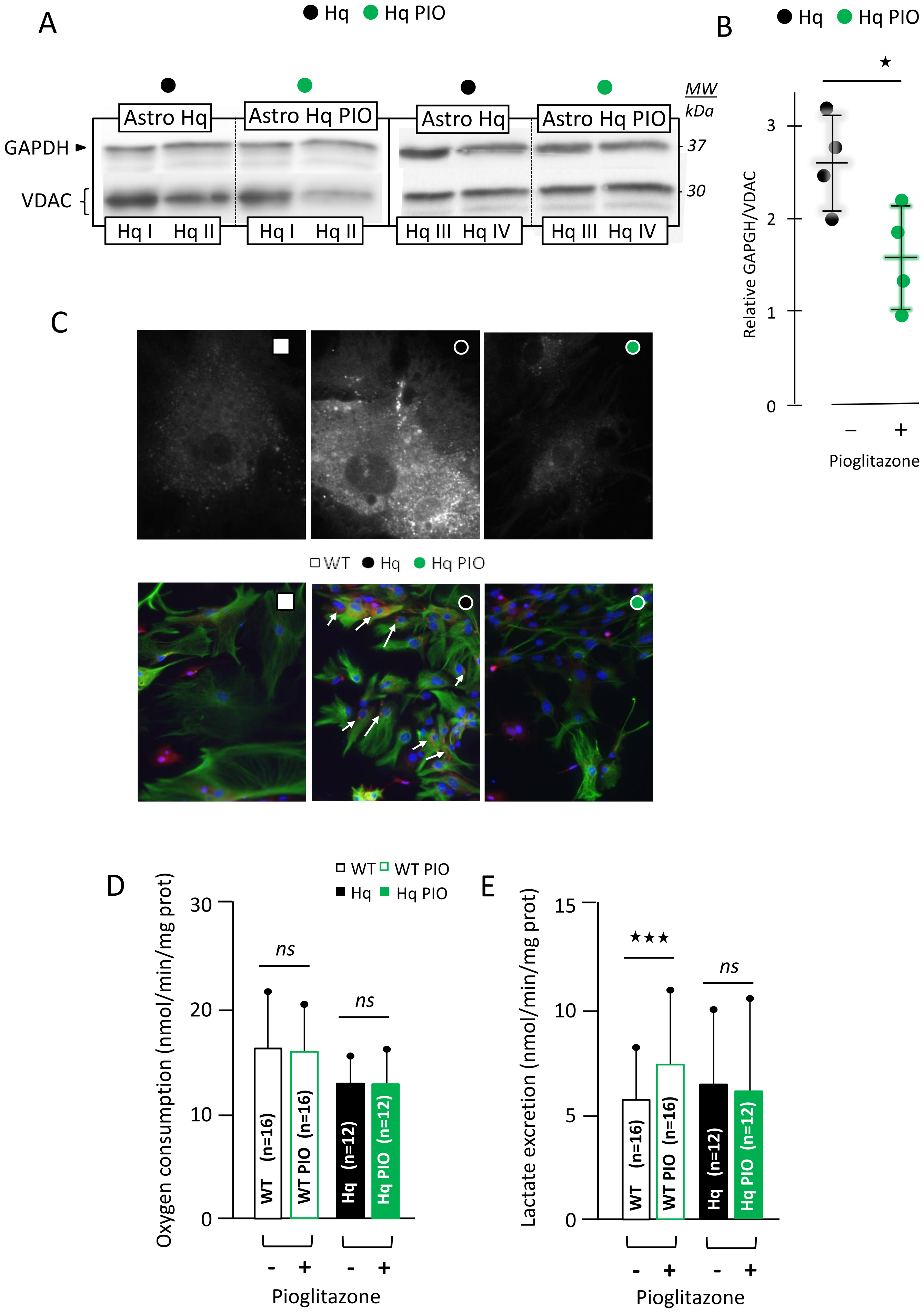
A
Hq
Hq PIO
Astro Hq
Astro Hq PIO
Astro Hq
Astro Hq PIO
MW
kDa
GAPDH
VDAC
37
30
Hq I Hq II
Hq I Hq II
Hq III Hq IV
Hq III Hq IV
B
Hq
Hq PIO
Relative GAPGH/VDAC
Pioglitazone
C
WT
Hq
Hq PIO
D
WT
WT PIO
Hq
Hq PIO
Oxygen consumption (nmol/min/mg prot)
ns
ns
WT (n=16)
WT PIO (n=16)
Hq (n=12)
Hq PIO (n=12)
Pioglitazone
E
Lactate excretion (nmol/min/mg prot)
★★★
ns
WT (n=16)
WT PIO (n=16)
Hq (n=12)
Hq PIO (n=12)
Pioglitazone

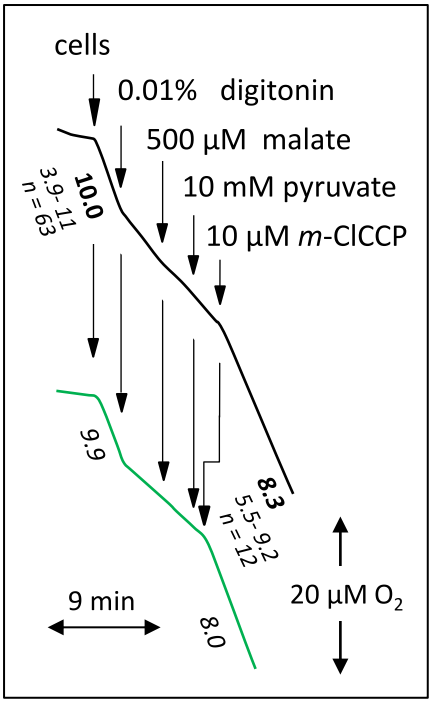

Bénit et al. Suppl Fig. 5

Table S1. Enzyme activities in cultured skin fibroblasts from AIF mutant patients and controls.

	Rotenone-sensitive NADH-dehydrogenase	Malonate-sensitive succinate dehydrogenase	Glycerol-3-phosphate dehydrogenase	Cytochrome c oxidase	Oligomycin-sensitive ATPase	Hexokinase	Glucose 6 phosphate dehydrogenase	Lactate dehydrogenase	Total superoxide dismutase
	(nmol/min/mg prot)								(IU/mg prot)
Fibroblasts									
P1 (2729)	4.2 ± 0.5	10.1 ± 1.5	7.4 ± 0.1	**4.1 ± 0.8**	64.2 ± 15.8	44.1 ± 2.1	135 ± 13	891 ± 395	2.8 ± 0.6
P2 (2732)	4.5 ± 0.6	11.5 ± 2.5	10.0 ± 2.5	**6.4 ± 1.0**	64.7 ±9.0	41.3 ± 6.8	111 ± 5	927 ± 362	3.0 ± 0.8
Control (n= 6)	6.2 ± 2.1	9.4 ± 2.6	9.8 ± 1.4	12.3 ± 3.8	54.3 ± 10.4	44.8 ± 5.0	135 ± 10	752 ± 184	4.8 ± 0.3
	*[1]	ns	ns	**	ns	ns	ns	ns	ns

[1]Signicance of difference between patients and controls
